# Supplementary material for: Erythroid lineage chromatin accessibility maps facilitate identification and validation of NFIX as a fetal hemoglobin repressor
Source: Commun Biol. 2023 Jun 14;6:640. doi: 10.1038/s42003-023-05025-4 (PMC10267139; doi:10.1038/s42003-023-05025-4)

Supplementary Information

Erythroid Lineage Chromatin Accessibility Maps Facilitate Identification and Validation of NFIX As a Fetal Hemoglobin Repressor

Mudit Chaand, Chris Fiore, Brian Johnston, Anthony D'Ippolito, Diane H Moon, John P Carulli, Jeffrey R Shearstone

Supplementary Figure 1

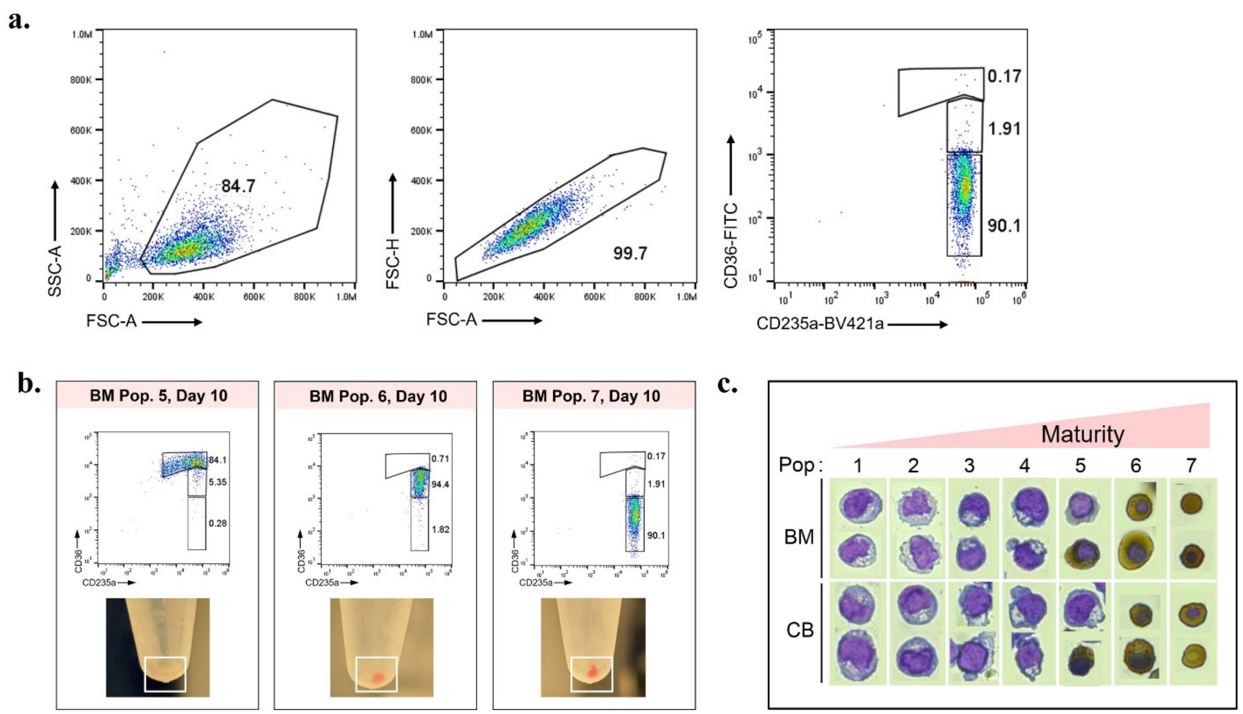

Supplementary Figure 1. Sorted cells resolve maturational changes obscured in a pooled approach. **a,** Example gating strategy (BM population 7) to determine purity of sorted cell populations. **b**, Sorted populations were analyzed by flow cytometry and visually by centrifugation. Cell populations sorted from BM progenitors on day 10 of erythroid differentiation are shown as an example. **c,** Cytospin analyses of sorted BM and CB cell populations to confirm their purity. All images were captured at 40X magnification using the Axio Lab.A1 light microscope (Zeiss). Data are representative of two biological replicates.

Supplementary Figure 2

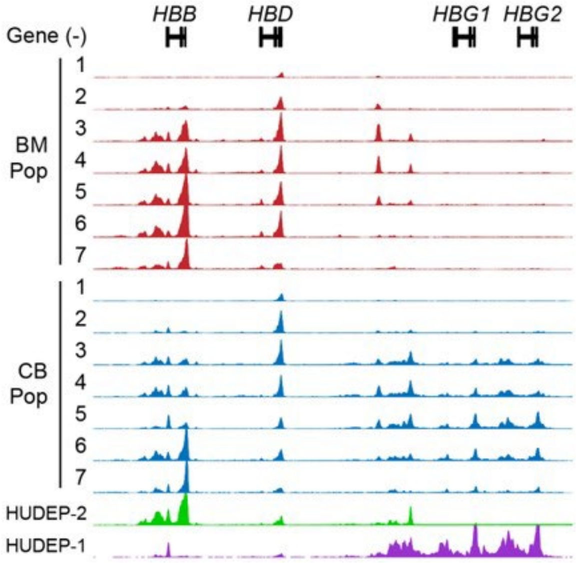

Supplementary Figure 2. BM and CB cells show distinct patterns of chromatin accessibility at the beta-like globin gene cluster. ATAC-seq profiles (spanning Chr11:5,245,000–5,277,000) of each sorted BM and CB population at the beta-like globin locus. HUDEP-1 and HUDEP-2 cells were included as additional HbF-high (fetal) and HbF-low (adult) controls, respectively. Data are representative of two biological replicates.

Supplementary Figure 3

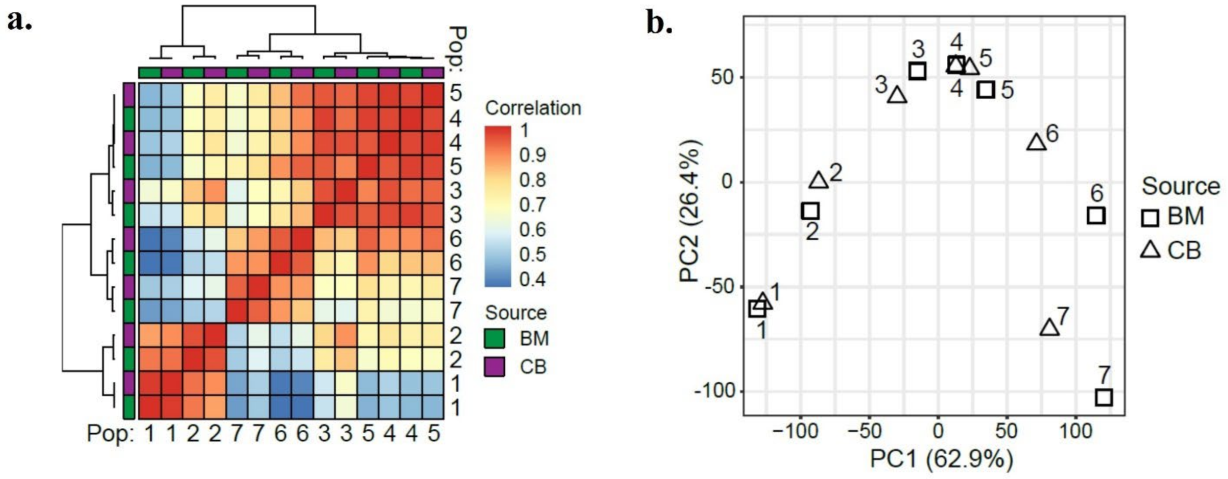

Supplementary Figure 3. The majority of chromatin accessibility differences in erythroid populations can be attributed to their maturational stage rather than their adult or fetal lineage of origin. a, Hierarchical clustering and **b,** Principal Component (PC) Analysis of ATAC-seq dataset from sorting of seven discrete cell populations from BM- and CB-derived progenitors. Data are representative of two biological replicates.

Supplementary Figure 4

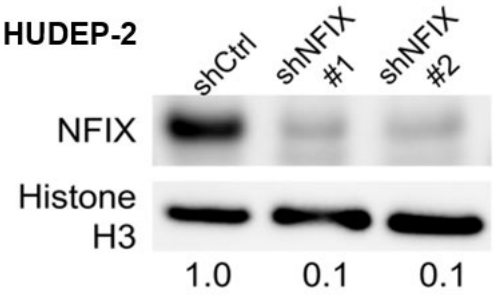

Supplementary Figure 4. shRNA-mediated knockdown of NFIX in HUDEP-2 cells leads to loss of NFIX protein. Western blot validation of NFIX knockdown in HUDEP-2 cells shows an approximately 90% reduction in NFIX protein relative to Histone H3 control. Hairpins that led to maximal knockdown of NFIX in BM cells (shNFIX #1 and shNFIX #2, Main Figure 3) were used to transduce HUDEP-2 cells**.** Data are representative of two independent HUDEP-2 transductions with lentivirus harboring shNFIX #1 or shNFIX #2.

Supplementary Figure 5

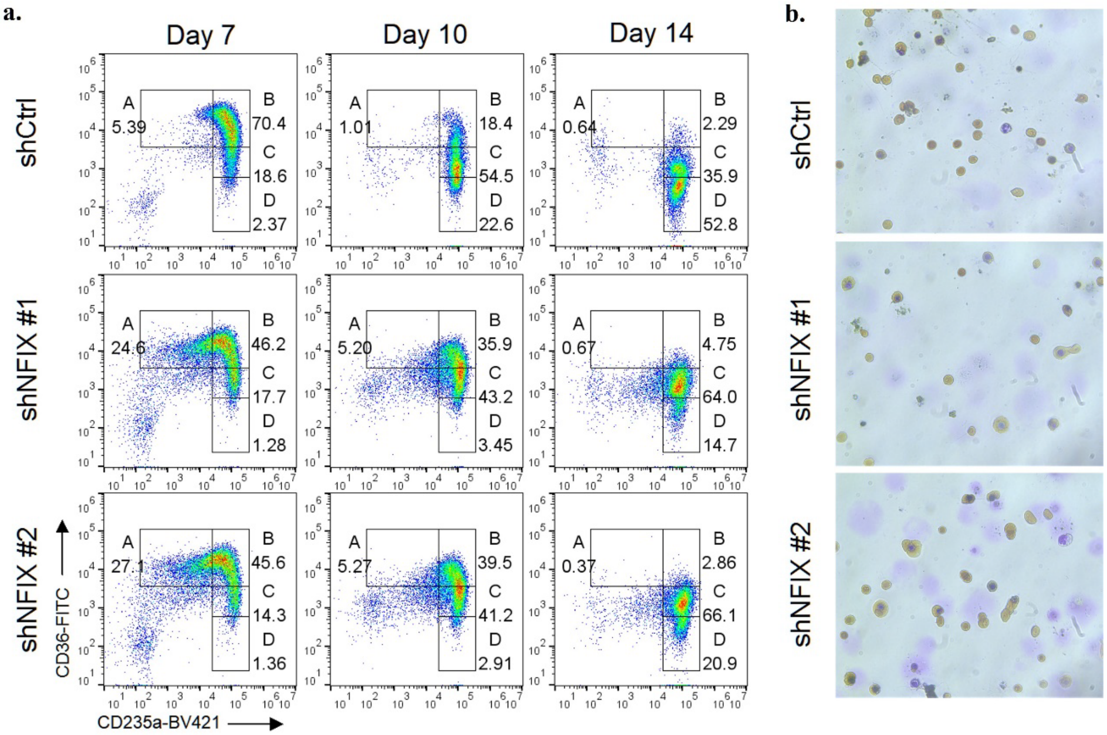

Supplementary Figure 5. NFIX knockdown cells can terminally differentiate. a, Erythroid surface marker (CD235a-BV421 and CD36-FITC) staining profiles of BM control (shCtrl) and NFIX knockdown cells (shNFIX #1-2) on days 7, 10 and 14 of erythroid differentiation indicate a slight delay in maturation of knockdown cells relative to the control. Gates C and D represent less mature and more mature reticulocytes, respectively. **b,** Representative cytospin images of control and NFIX knockdown cells show hemoglobinized and enucleated reticulocytes on day 14. Data shown are representative of three biological replicates from distinct donors.

Supplementary Figure 6

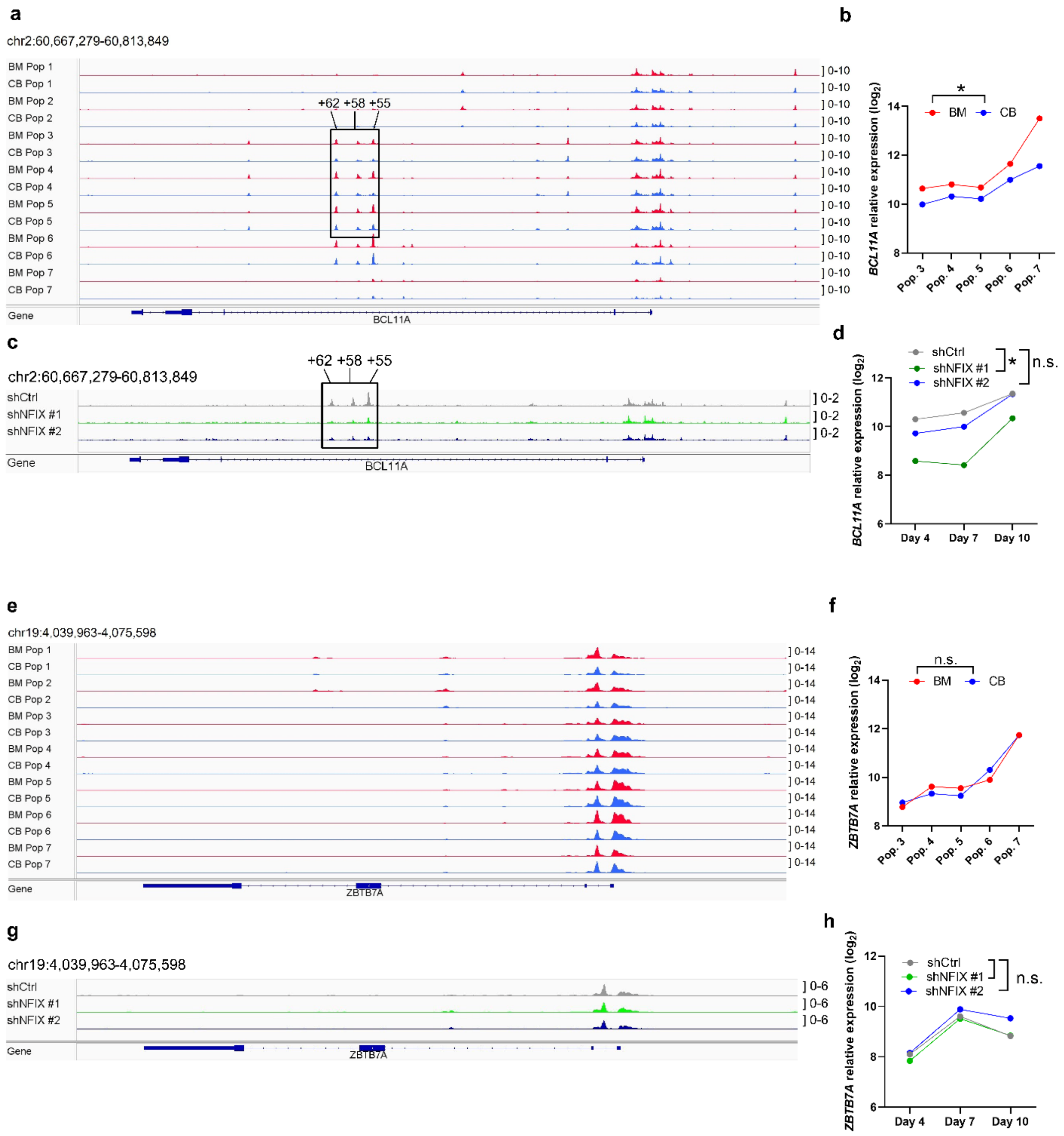

Supplementary Figure 6. Cells with lower levels of NFIX show reduced chromatin accessibility at the BCL11A enhancer and lower levels of BCL11A mRNA. a, ATAC-seq profiles of sorted BM and CB populations at the *BCL11A* locus showing lower chromatin accessibility in populations 3-5 of CB cells (lower NFIX) relative to BM (higher NFIX). Intronic *BCL11A* erythroid-specific enhancers at +55

kb, +58 kb and +62 kb relative to the transcription start site are boxed. **b,** Corresponding mRNA samples show reduced BCL11A expression levels in CB. **c,** ATAC-seq profiles of NFIX knockdown BM cells at day 4 of differentiation show reduced chromatin accessibility at the *BCL11A* enhancer (boxed) upon NFIX knockdown relative to control. **d,** Matched mRNA samples from NFIX knockdown in BM cells on days 4, 7 and 10 of differentiation show reduced *BCL11A* levels relative to control. **e-h,** *ZBTB7A* chromatin accessibility profiles and mRNA levels in the same experiments as described in **a-d**. ATAC-seq and RNA-seq data derived from same biological replicate. Statistical significance was determined using a Student's *t*-test paired by population or culture day. Asterisks denote $P < 0.05$; n.s., not significant.

Supplementary Figure 7. Uncropped Western blot images with corresponding figure numbers.

Uncropped Figure 2d

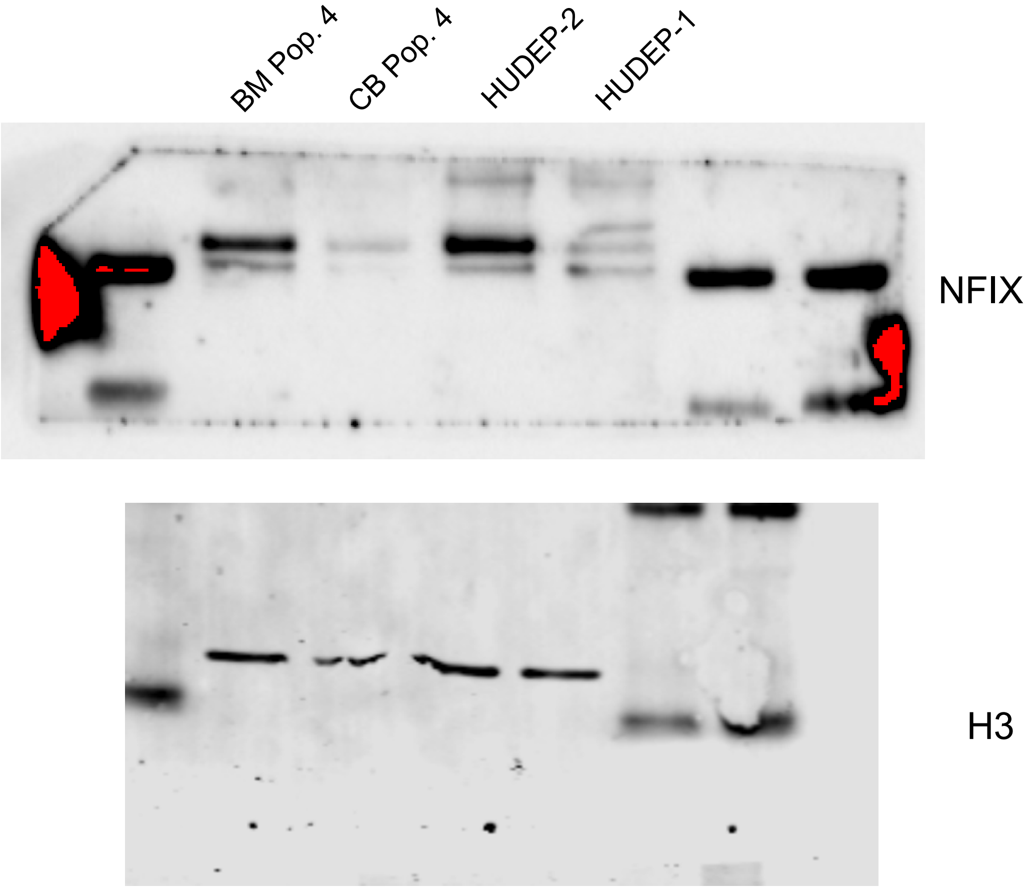

Uncropped Figure 4b

Predicted size of NFIX-FLAG: 53 kDa

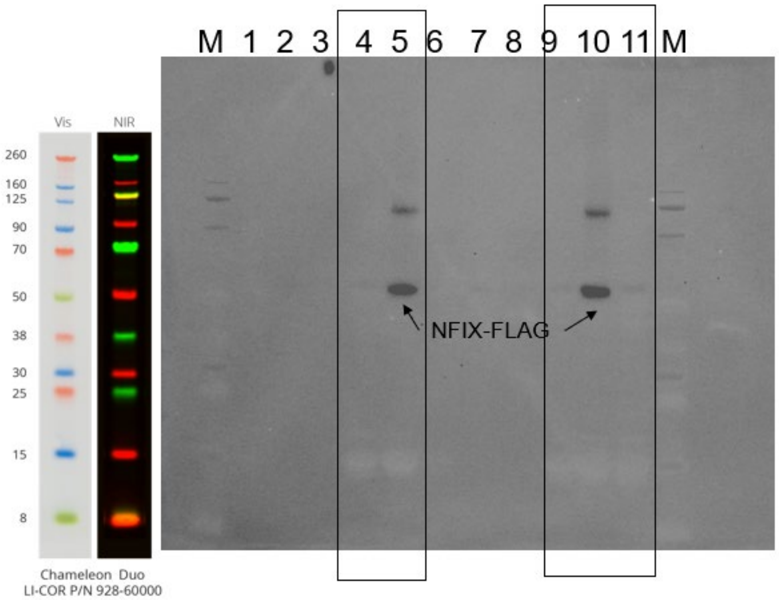

Lane	Sample
M	Ladder
1	
2	
3	
4	Day 14 Empty
5	Day 14 NFIX
6	
7	
8	
9	Day 14 Empty
10	Day 14 NFIX
11	Day 14 Empty
M	Ladder

Loading control- GAPDH, 36 kDa

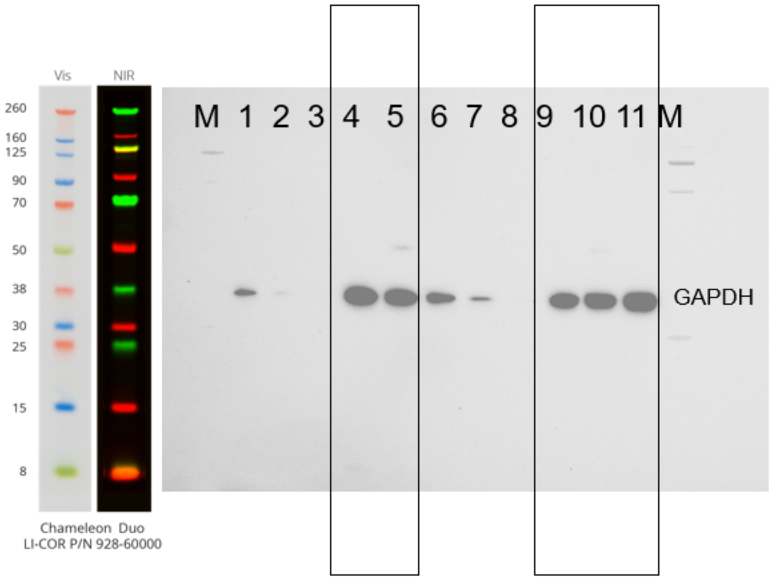

Lane	Sample
M	Ladder
1	
2	
3	
4	Day 14 Empty
5	Day 14 NFIX
6	
7	
8	
9	Day 14 Empty
10	Day 14 NFIX
11	Day 14 Empty
M	Ladder

Uncropped Supplementary Figure 4

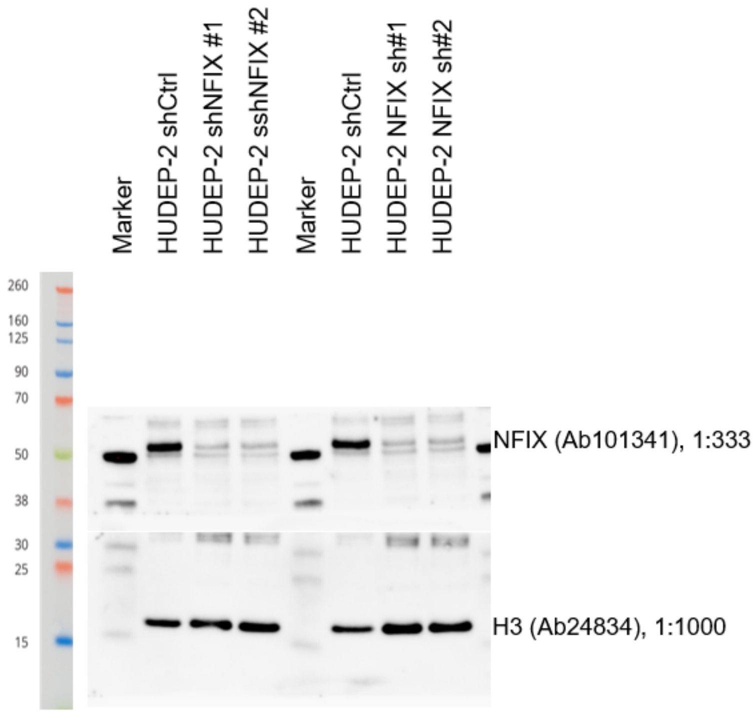

Supplement: Supplementary file 2 — Supplementary Information [file 42003_2023_5025_MOESM2_ESM.pdf]
