# Supplementary material for: Erythroid lineage chromatin accessibility maps facilitate identification and validation of NFIX as a fetal hemoglobin repressor
Source: Commun Biol. 2023 Jun 14;6:640. doi: 10.1038/s42003-023-05025-4 (PMC10267139; doi:10.1038/s42003-023-05025-4)
Supplement: Supplementary file 3 — Description of Additional Supplementary Files [file 42003_2023_5025_MOESM3_ESM.pdf]

Description of Additional Supplementary Files

File name: Supplementary Data 1

Description: ATAC-seq BM vs. CB enrichment.

File name: Supplementary Data 2

Description: The source data behind the plots and graphs in the paper.
