# Supplementary material for: Erythroid lineage chromatin accessibility maps facilitate identification and validation of NFIX as a fetal hemoglobin repressor
Source: Commun Biol. 2023 Jun 14;6:640. doi: 10.1038/s42003-023-05025-4 (PMC10267139; doi:10.1038/s42003-023-05025-4)
Supplement: Supplementary file 6 — Reporting Summary [file 42003_2023_5025_MOESM6_ESM.pdf]

nature portfolio

Corresponding author(s): Mudit Chaand

Last updated by author(s): 06/02/2023

Reporting Summary

Nature Portfolio wishes to improve the reproducibility of the work that we publish. This form provides structure for consistency and transparency in reporting. For further information on Nature Portfolio policies, see our Editorial Policies and the Editorial Policy Checklist.

Statistics

For all statistical analyses, confirm that the following items are present in the figure legend, table legend, main text, or Methods section.

n/a	Confirmed	
☐	☒	The exact sample size (n) for each experimental group/condition, given as a discrete number and unit of measurement
☐	☒	A statement on whether measurements were taken from distinct samples or whether the same sample was measured repeatedly
☐	☒	The statistical test(s) used AND whether they are one- or two-sided<br>*Only common tests should be described solely by name; describe more complex techniques in the Methods section.*
☐	☒	A description of all covariates tested
☐	☒	A description of any assumptions or corrections, such as tests of normality and adjustment for multiple comparisons
☐	☒	A full description of the statistical parameters including central tendency (e.g. means) or other basic estimates (e.g. regression coefficient) AND variation (e.g. standard deviation) or associated estimates of uncertainty (e.g. confidence intervals)
☐	☒	For null hypothesis testing, the test statistic (e.g. F, t, r) with confidence intervals, effect sizes, degrees of freedom and P value noted<br>*Give P values as exact values whenever suitable.*
☒	☐	For Bayesian analysis, information on the choice of priors and Markov chain Monte Carlo settings
☒	☐	For hierarchical and complex designs, identification of the appropriate level for tests and full reporting of outcomes
☐	☒	Estimates of effect sizes (e.g. Cohen's d, Pearson's r), indicating how they were calculated

Our web collection on statistics for biologists contains articles on many of the points above.

Software and code

Policy information about availability of computer code

Data collection	Data collection for high-throughput DNA sequencing data was collected on a Illumina HiSeq2500 instrument using HCS (v2.2.70).
Data analysis	All analysis of high-throughput DNA sequencing data was performed in R (v4.1.1) using the software packages cited in the manuscript. Code for custom analyses is available upon request.

For manuscripts utilizing custom algorithms or software that are central to the research but not yet described in published literature, software must be made available to editors and reviewers. We strongly encourage code deposition in a community repository (e.g. GitHub). See the Nature Portfolio guidelines for submitting code & software for further information.

Data

Policy information about availability of data

All manuscripts must include a data availability statement. This statement should provide the following information, where applicable:

- Accession codes, unique identifiers, or web links for publicly available datasets
- A description of any restrictions on data availability
- For clinical datasets or third party data, please ensure that the statement adheres to our policy

Raw and processed sequencing data generated in this study are available from the Gene Expression Omnibus under accession ***.

Field-specific reporting

Please select the one below that is the best fit for your research. If you are not sure, read the appropriate sections before making your selection.

☒ Life sciences ☐ Behavioural & social sciences ☐ Ecological, evolutionary & environmental sciences

For a reference copy of the document with all sections, see nature.com/documents/nr-reporting-summary-flat.pdf

Life sciences study design

All studies must disclose on these points even when the disclosure is negative.

Sample size	Comparative ATAC-seq studies were performed using CD34+ hematopoietic stem cells (HSCs) from a single bone marrow (BM) donor and pooled HSCs from six cord blood (CB) donors. For NFIX knockdown studies, three independent biological replicates were performed using BM CD34+ HSCs. Two independent biological replicates were performed for NFIX overexpression studies using pooled CB CD34+ HSCs from multiple donors. Distinct BM and CB CD34+ HSC vials were commercially sourced for all experiments listed above. Samples were collected at different time points of erythroid differentiation and days of collection are stated in the figures and /or figure legends.
Data exclusions	None.
Replication	Data were reliably reproduced.
Randomization	None.
Blinding	None.

Reporting for specific materials, systems and methods

We require information from authors about some types of materials, experimental systems and methods used in many studies. Here, indicate whether each material, system or method listed is relevant to your study. If you are not sure if a list item applies to your research, read the appropriate section before selecting a response.

Materials & experimental systems

n/a	Involved in the study	
☐	☒	Antibodies
☐	☒	Eukaryotic cell lines
☒	☐	Palaeontology and archaeology
☒	☐	Animals and other organisms
☒	☐	Human research participants
☒	☐	Clinical data
☒	☐	Dual use research of concern

Methods

n/a	Involved in the study	
☒	☐	ChIP-seq
☐	☒	Flow cytometry
☒	☐	MRI-based neuroimaging

Antibodies

Antibodies used	Anti-NFIX (Abcam- Ab101341, lot GR3173994-11, 1:1000 dilution)<br>Anti-H3 (Abcam Ab24834, lot GR3187597-1, 1:1000 dilution)<br>Anti-GAPDH-HRP (Abcam Ab97051, lot GR3342710-9, 1:2000 dilution)<br>Anti-BCL11A (Abcam Ab 191401, lot GR3260079-3, 1:500 dilution)<br>Anti-LRF (custom monoclonal antibody, Genscript, 1:3000)<br>Anti-HbF-FITC (Thermo Fisher Scientific, MHFH01, multiple lots, 10 µL/test for up to 1 million cells)<br>Anti-Mouse IgG1 FITC (BD Biosciences, clone X40, multiple lots, 40 µL/test for up to 1 million cells)<br>Anti-CD36-FITC (BD Biosciences 555454, multiple lots, 20 µL/test for up to 1 million cells)<br>Anti-CD71-APC (BD Biosciences 551374, multiple lots, 20 µL/test for up to 1 million cells)<br>Anti-CD235a-BV421 (BD Biosciences 562938 clone GA-R2 (HIR2), multiple lots, 5 µL/test for up to 1 million cells)
Validation	All antibodies (except anti-LRF) were commercially sourced after reviewing validation summaries listed on the vendor websites. All antibodies used in this study have also been previously reported in the literature and are commonly used in red blood cell studies. The anti-LRF antibody was custom made by Genscript for internal use at Syros Pharmaceuticals and was validated extensively in-house.

March 2021

Eukaryotic cell lines

Policy information about cell lines

Cell line source(s)	Human Umbilical cord Derived Erythroid Progenitor (HUDEP)- 1 and 2 cells were purchased under a licensing agreement from RIKEN BioResource Research Center, Japan. The cell lines were first reported in Kurita et al., PLoS One 2013 PMID: 23533656. BM and CB CD34+ HSCs were purchased from AllCells, LLC and were >90% pure as indicated on the certificates of analysis obtained for each vial from the supplier.
Authentication	HUDEP-1 and HUDEP-2 cells were authenticated by performing RT-qPCR on human globin genes and quantifying HbF by flow cytometry and HPLC to confirm their HbF-high and HbF-low phenotypes, respectively. Chromatin accessibility profiles at the beta-like globin gene cluster obtained from these cell lines in the ATAC-seq experiments reported in this study further confirmed their authenticity.
Mycoplasma contamination	All cell lines used in the study were routinely tested for Mycoplasma contamination using a PCR-based test from ATCC (catalog no. 30-1012K).
Commonly misidentified lines (See ICLAC register)	None.

Flow Cytometry

Plots

Confirm that:

☒ The axis labels state the marker and fluorochrome used (e.g. CD4-FITC).

☒ The axis scales are clearly visible. Include numbers along axes only for bottom left plot of group (a 'group' is an analysis of identical markers).

☒ All plots are contour plots with outliers or pseudocolor plots.

☒ A numerical value for number of cells or percentage (with statistics) is provided.

Methodology

Sample preparation	F-cell assays: Primary BM or CB cells, HUDEP-1 and HUDEP-2 cells were fixed with 0.05 % glutaraldehyde (Sigma) in PBS for 10 min at room temperature and centrifuged at 600 x g for 5 min. Fixed cells were washed three times in FACS buffer (PBS + 0.5% BSA) and permeabilized with Triton X-100 (Life Technologies) for 5 min at room temperature. Fixed and permeabilized cells were stained with appropriate antibodies (detailed in the text and the reporting summary) in the dark at 4 C for 30 min. Cells were then washed with FACS buffer and resuspended in FACS buffer prior to running on the instrument listed below.<br>Erythroid surface marker staining: Live cells were co-stained with appropriate antibodies (detailed in the text and the reporting summary) in the dark at 4 C for 30 min. Cells were then washed with FACS buffer and resuspended in FACS buffer prior to running on the instrument below.
Instrument	Sony SH800 cell sorter (Sony Biotechnology)
Software	FlowJo™ (Becton, Dickinson and Company; 2019)
Cell population abundance	10-20,000 sorted cells were run on the Sony SH800 to confirm purity of the sorted BM and CB populations (>95%).
Gating strategy	F-cell assays: cells were gated in the following sequence (X and Y axes): 1. viability (FSC-A and SSC-A), 2. single cells (FSC-A and FSC-H), 3. HbF+ cells (FSC-A and FITC-A). FITC mouse IgG1 was used as an isotype control antibody to set the HbF+ gate at ~1%.<br>Erythroid marker staining: cells were gated in the following sequence (X and Y axes): 1. viability (FSC-A and SSC-A), 2. single cells (FSC-A and FSC-H), 3. population of interest (BV421-A and FITC-A). Unstained and single-color stained cells were used as controls to set up the compensation matrix to minimize spectral overlap.<br>Supplementary Figure 1a exemplifies the gating strategy.

☒ Tick this box to confirm that a figure exemplifying the gating strategy is provided in the Supplementary Information.
